# Relationship between Metal Pollution and Gene Expression of Insulin-like Growth Factor II

**DOI:** 10.5696/2156-9614-8.18.180608

**Published:** 2018-06-11

**Authors:** Aziza A. Saad, Amany El-Sikaily, Maher A. Kamel, Hany Kassem, Mohamed S. Abdel-Latif

**Affiliations:** 1. Applied Medical Chemistry, Medical Research Institute, Alexandria University; 2. Marine Pollution Department, National Institute of Oceanography & Fisheries, Ministry of Scientific Research, Egypt; 3. Biochemistry Department, Medical Research Institute, Alexandria University; 4. Department of Medical Laboratory Technology, Faculty of Allied Medical Science, Pharos University in Alexandria, Egypt

**Keywords:** heavy metal contamination, insulin-like growth factor II, glutathione peroxidase

## Abstract

**Background.:**

Metals pollution plays an important role in the regulation of gene expression through interference with signal transduction pathways which are important for cell bioactivity.

**Objectives.:**

The present study was conducted to estimate metallothionein levels in mussels as a biomarker of exposure to heavy metals in order to monitor the pollution of Abu Qir Bay, Egypt (El-Maadiya region) and to evaluate the impact of heavy metals on human health by examining insulin-like growth factor II (IGF-2) gene expression in peripheral blood mononuclear cells.

**Methods.:**

One hundred and forty mussel samples (*Andara dulofii*) were collected from Abu-Qir Bay, stored in bags, preserved in an ice box, and then transported to the laboratory to acclimatize at 20°C for three days in ethylene diamine tetra acetic acid (EDTA)-free synthetic sea water to determine the presence of metallothionein and five other metals (cadmium (Cd), lead (Pb), chromium (Cr), copper (Cu) and zinc (Zn)).

**Results.:**

Results showed that mussels collected from the study area contained a measurable amount of metallothionein. In addition, results revealed an increased level of malondialdehyde coinciding with a decreased level of antioxidants, leading to oxidative stress in local fishermen.

**Conclusions.:**

The present data demonstrated a significant increase in the gene expression of IGF-2 and a positive correlation between IGF-2 gene expression and the enzymatic activity of glutathione peroxidase in male subjects.

**Participant Consent.:**

Obtained

**Ethics Approval.:**

Written consent was provided by the study participants and study approval was given by the ethics committee of Alexandria University (US Department of Health and Human Services, Registration of an Institutional Review Board, IORG0008812 Medical Research Institute, Expires 4/8/2019, OMB No:0990-0279).

**Competing Interests.:**

The authors declare no competing financial interests

## Introduction

Metals play an important role in many biological processes of living systems. Homeostasis of metal ions maintained by regulated mechanisms of uptake, storage and secretion is essential for life.^1^ Carriers for various metal ions participate in maintaining their intracellular basal levels.^2^ Essential transition metals such as zinc (Zn), iron (Fe), copper (Cu), cobalt (Co) and manganese (Mn) play a substantial role in the control of various metabolic and signaling pathways. Metals pollution plays an important role in the regulation of gene expression through interference with signal transduction pathways important for cell bioactivity.^3^

Impairment of cell growth and differentiation is a feature of cancer development, where metals interfere with cell proliferation by activating various transcription factors that control cell growth and apoptosis.^4^ In addition, the mechanism involves formation of a superoxide radical, hydroxyl radical, and finally the production of mutagenic and carcinogenic malondialdehyde (MDA), 4-hydroxynonenal, and exocyclic DNA (deoxyribonucleic acid) adducts.^5^ Carcinogenic metals and metalloids including arsenic, cadmium, nickel and cobalt have the capacity to inhibit zinc fingers in DNA repair proteins.^6^

Contamination of aquatic environments with heavy metals can be estimated by analyzing water, sediments and marine organisms. It has been shown that levels of heavy metals are often higher in mollusks and other invertebrates than in other constituents of the aquatic environment due to their habitat and feeding habits.^7–9^ Furthermore, mollusks are more susceptible to heavy metal accumulation than sediments and are considered to be a source of metals contamination.^10–13^ Exposure to heavy metals has a harmful effect on human health and development. Epigenetic changes resulting from exposure to heavy metals could be an inducer of these harmful effects. In addition, regulation of gene transcription may be affected by epigenetic changes that can be established and maintained in DNA through methylation.^14^ Regulation of gene transcription is important for growth and energy utilization. Insulin-like growth factor II (IGF-2) is a good example of regulation of gene transcription which is regulated by at least two differentially methylated regions. Exposure of human cells to heavy metals such as lead (Pb) changes both IGF-2 gene expression and DNA methylation at the differentially methylated regions.^14^ Epigenetic alteration of IGF-2 by methylation has been associated with obesity, neurodevelopmental disorders, and cancer.^14^

The present study was conducted to estimate metallothionein levels in mussels as a biomarker of exposure to heavy metals in order to monitor the pollution of Abu Qir Bay, Egypt (El-Maadiya region) and to examine the impact of heavy metals on human health through the study of IGF-2 gene expression in peripheral blood mononuclear cells.

## Methods

### Study area

**Figure 1:**
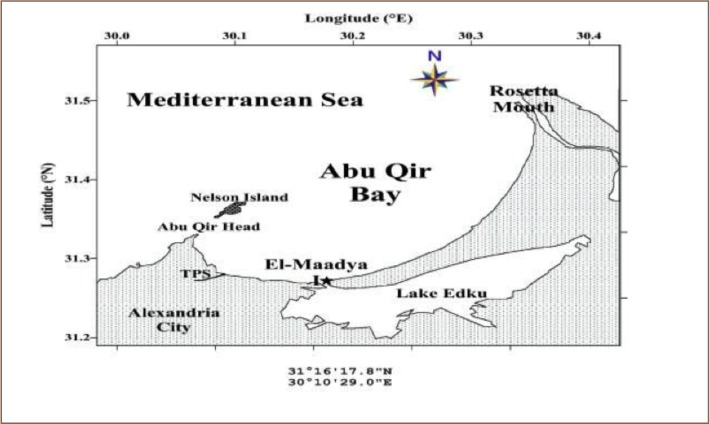
Map of study area

Abu Qir Bay is a semicircular shallow basin about 35 km east of Alexandria between Abu Qir Peninsula (west) and the Rosetta branch of the Nile (east), with a shore line extending about 50 km (*Figure 1*).^15^ It lies between 30°4′–30 21′ East and 31°16′–31 30′ North. It is relatively shallow with a depth ranging from less than 1 m along the coast, gradually increasing away from the shore to reach a maximum depth of about 15 m. In Abu Qir Bay, large amounts of industrial wastes are discharged into the bay through the Tabia pumping station.

**Abbreviations**
*EDTA*: Ethylene diamine tetra acetic acid
*GAPDH*: Glyceraldehyde-3-phosphate dehydrogenase
*GPx*: Glutathione peroxidase
*IGF-2*: Insulin-like growth factor II
*MDA*: Malondialdehyde
*PCR*: Polymerase chain reaction
*RNA*: Ribonucleic acid
*SD*: Standard deviation

### Biological matrix

One hundred and forty mussel samples (*Andara dulofii*) were collected from the Mediterranean Sea, Abu Qir Bay, in the El-Maadiya region. They were stored in bags and preserved in an ice box, then transported to the laboratory to acclimatize to conditions at 20°C for three days in EDTA-free synthetic sea water (pH 7.9–8.0 and 35 osmolarity and salinity) to determine the presence of metallothionein and five other metals (Cd, Pb, Cr, Cu and Zn).^16^,^17^

### Blood samples

A total of 50 male subjects, ranging in age from 20 – 55 years old and weight range 62–85 kg were divided into two groups.

Control (Group I): Included 10 control subjects; healthy control males living in the El-Maadiya region but working in jobs other than fishing.

Fishermen (Group II): Included 40 professional fishermen living in the El-Maadiya region. Information on age, weight, area of residence, smoking history, caffeine consumption, medication used and history of acute or chronic illness were gathered via questionnaire. The food habits of study subjects were also taken into consideration. Subjects with history of smoking, thyroid dysfunction, diabetes, or liver disease were excluded.

Venous blood was collected from all enrolled subjects for the assay of heavy metals (Cd, Pb, Cr, Cu and Zn), MDA, glutathione peroxidase (GPx) activity, and ribonucleic acid (RNA) was extracted from peripheral blood mononuclear cells with a commercially available kit for the study of IGF-2 expression level.^18–22^ Special vacutainer tubes (EDTA, royal bluetop) were used for collecting venous blood for the assay of heavy metals.

### Chemicals

All chemicals used in the present study such as absolute ethanol, chloroform, hydrochloric acid and nitric acid were of high performance liquid chromatography grade. In addition, sodium citrate, 5, 5′-dithiobis-2-nitrobenzoic acid, phenyl methyl sulfonyl fluoride, leupeptin, thiobarbituric acid, and reduced glutathione were from Millipore Sigma, and potassium hydroxide, sodium hydroxide and sodium chloride were from Al-Nasr Pharmaceutical Chemicals Company (Al-Dawa City, ADWIC, Egypt). IQ easy Plus Blood RNA Extraction Kit and Maxime Reverse Transcription Polymerase Chain Reaction (RT-PCR) Premix were from iNtRON Biotechnology (Korea). DNA primer sequences were from Jena BioScience (Germany). PCR Master Mix, magnesium chloride and polymerase chain reaction (PCR) markers were from Promega (USA).

### Ethics approval

Written consent was provided by the study participants and study approval was given by the ethics committee of Alexandria University (US Department of Health and Human Services, Registration of an Institutional Review Board, IORG0008812 Medical Research Institute, Expires 4/8/2019, OMB No: 0990-0279).

### Determination of mussel metallothionein

Metallothionein concentration was evaluated using a partially purified metalloprotein fraction obtained by acidic ethanol/chloroform fractionation of the tissue homogenate and measured colorimetrically using glutathione (GSH).^16^

### Determination of heavy metals in mussels

Mussel gills and digestive glands were rapidly dissected using Teflon^®^ knives. Fourteen composite samples of mussel tissues (10 mussels of similar size/each composite sample) were homogenized with an Ultra-Turrax T25 homogenizer. To avoid contamination, all parts of the homogenizer coming into contact with the samples were covered with Teflon^®^ adaptors. The 14 homogenated composite samples were stored at −80°C for determination of metals content (Cd, Pb, Cr, Cu, and Zn). All digested solutions were analyzed and measured using an atomic absorption spectrophotometer (SPECTR plus version) with an air-acetylene flame and deuterium background correction.^17^ Method accuracy was verified using a standard reference material (freeze-dried mussel tissues, *Mytilus edulis*; IAEA-433, International Atomic Energy Agency; Analytical Quality Control Services).

### Quality control

All the plasticware and Teflon cups were previously soaked overnight with 10% nitric acid and then rinsed. Each element concentration was estimated quantitatively according to the standard conditions described in the instrument manual. However, working standards of studied elements were prepared by diluting concentrated stock solutions with deionized water. Reagents of analytical grade were utilized for the blanks and calibration curves, and precision was checked against a standard reference material (IAEA-433 for mussels, International Atomic Energy Agency; Analytical Quality Control Services and National Institute of Standard and Technology SRM 955c for whole blood) which was analyzed with either a solution of digested mussels or human whole blood during the course of analysis. The measured concentrations of heavy metals for mussels and whole blood were within the range of certified values with a recovery of 96.9–105.5%, and precision was within 10% and the standard deviations (SD) were low (range 0.7% to 1.1%), ensuring a high reproducibility of the method. The mean recoveries of studied metals for four replicates of each standard reference material were 105.5± 1.1% for Cd; 96.9± 0.7% for Cu; 105.2± 0.97% for Cr; and 98.0± 0.81% for Zn. Interestingly, each of the standard reference materials were similar in term of the absorption wave length and detection limits of heavy metals at 228.8 nm and 0.006 μg/g for Cd; 324.7 nm and 0.008 μg/g for Cu; 273.9 nm and 0.005 μg/g for Cr; 213.9 nm; and 0.004 μg/g for Zn.

### Heavy metal detection limits

Two concentrations of each element were prepared using a stock standard (1000 mg/L single element standard, purchased from Perkin Elmer), with entirely separate volumetric plasticware for each standard concentration to reduce the possibility of contamination. The lower concentration standard was approximately 5x the expected detection limit, and the second standard was twice this concentration. Ten readings were taken for each standard. A reading of the blank (the entire reagent used in the sample preparation) was made between each standard reading. The average of the two blank readings that were taken immediately before and after each standard was calculated and subtracted from the standard reading. In addition, the mean and SD for the set of readings of each standard were calculated.

### Biochemical studies

#### Determination of blood heavy metals

Atomic absorption spectroscopy assay was used to determine the concentration of Cd, Pb, Cr, Cu, and Zn in whole blood samples of subjects. Blood metal concentrations were obtained from the standard curve and ranged from 0.25 to 5 mg/l (range of detection).^18^

#### Determination of serum lipid peroxide (malondialdehyde)

The principle of this procedure is based on the reaction of lipid peroxides (MDA) with thiobarbituric acid in an acidic medium forming a red pigment extracted using n-butanol and measured at 530 nm. The concentration of MDA was expressed as nmol/ml.^19^

#### Determination of erythrocytes glutathione peroxidase activity

Glutathione peroxidase activity was measured indirectly by a coupled reaction with glutathione reductase and oxidized glutathione, and the rate of decrease in the absorbance at 340 nm was directly proportional to GPx activity in the sample.^20^

#### Determination of erythrocytes superoxide dismutase activity

The superoxide dismutase activity assay was based on the ability of the enzyme to inhibit the auto-oxidation of 10 mM pyrogallol-hydrochloric acid solution.^21^

#### Hemoglobin assay

EDTA blood was centrifuged, then the plasma and leukocyte layer were discarded. The erythrocytes were washed three times with isotonic sodium chloride. Then 1% erythrocytes lysate was prepared using deionized water (10 ml erythrocytes + 990 ml deionized water). Drabkin's method was used to determine hemoglobin concentration of the prepared lysate.^22^

### Insulin-like growth factor II gene expression

Venous blood samples were withdrawn in sterile evacuated tubes containing EDTA as an anticoagulant and total RNA was extracted from peripheral blood mononuclear cells by RNeasy Mini Spin Column (Qiagen, USA) according to the manufacturer's instructions. RNA integrity was assessed by agarose gel electrophoresis. Purified RNA was converted into cDNA by RT-PCR using commercially available RT-PCR Premix (BIORON GmbH, Germany).

The resulting cDNA was amplified by a nested PCR with two pairs of primers. The oligonucleotides were designed according to IGF-2 gene sequence and synthesized by Jena Bioscience GmbH, Germany. The sequence of the two external primer pairs used for the initial PCR amplification was IGF-2-I (sense), 5′-ATGGGAATGCCAATGGGGAAG-3′ (nt 251–271) and IGF-2-II (antisense), 5′-CTTGCCCACGGGGTATCTGGG-3′ (nt 566–586). The size of the amplified gene fragment was 336 bp.^23^

The sequence of the two internal primer pairs used for the second PCR amplification was IGF-2-III (sense), 5′-TGCTGCATTGCTGCTTACCG-3′ (nt 311–330) and IGF-2-IV (antisense), 5′-AGGTCACAGCTGCGGAAACA-3′ (nt 461–480). The final product of nested PCR was 170 bp.^23^

Human glyceraldehyde-3-phosphate dehydrogenase (GAPDH) genome was used as a control. The primer sequence for GAPDH was GAPDH-1 (sense), 5′- ACCACAGTCCATGCCATCAC-3′ (nt 601–620) and GAPDH-2 (antisense), 5′- TCCACCACCCTGTTGCTGT A-3′ (nt 1033–1052), the product of PCR was 452 bp (GAPDH gene transcript, 40 pmol/L).

The PCR products were electrophoresed on 2% agarose gel with ethidium bromide staining. The fragment sizes were evaluated using PCR markers (Promega) as molecular weight standards.

Bands were scanned and the data were analyzed using UVP-DOC-ITLSTM Image acquisition and analysis software (Ultra Violet Product, Ltd, Cambridge, UK) to analyze the relative band intensities of IGF-2 to the GAPDH band (as internal control).

### Statistical data analysis

Data in the current study were statistically analyzed using SPSS version 20 (SPSS Inc., Chicago, IL, USA). Numerical data were expressed as mean ± SD, and the values for different variables of fishermen and control subjects were compared using the t-test for independent variance and all showed normal distribution (blood metals, malondialdehyde, glutathione peroxidase, superoxide dismutase and IGF-2 gene expression levels). The correlation analysis between different parameters was performed by Pearson's linear regression analysis. A two-sided p-value of < 0.05 was considered statistically significant.

## Results

### Metallothionein and heavy metals assay of mussels

**Figure 2:**
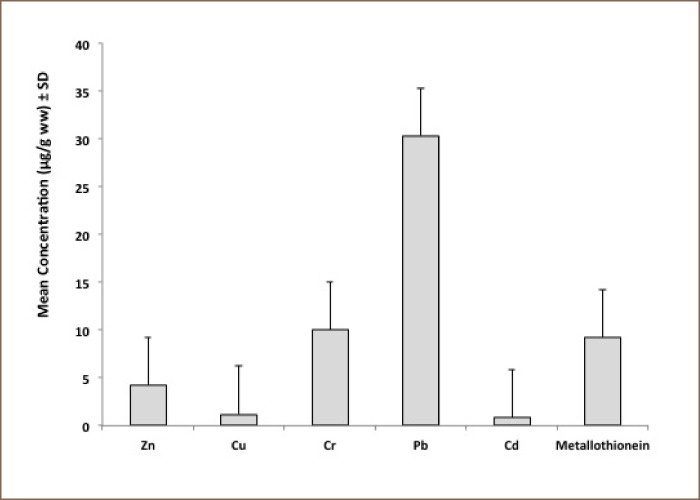
Metallothionein and metals mean concentrations in mussels collected from El-Maadiya region of the Mediterranean Sea (n = 14 composite samples)

Figure 2 presents the mean concentrations of metallothionein in 14 composite samples of mussels collected from the El-Maadiya region of the Mediterranean Sea (9.23 ± 1.94 μg/g wet weight). The mean concentrations of the five studied metals, Cd, Pb, Cr, Cu, and Zn in mussels were 0.822 ± 0.046, 30.29 ± 7.69, 9.99 ± 0.347, 1.195 ± 0.094, and 4.25 ± 0.576 μg/g wet weight, respectively (*Figure 2*). Levels of metallothionein and heavy metals in mussels from the contaminated area were compared to those obtained from the standard reference material. Levels of metallothionein and heavy metals in the standard reference material were lower than in contaminated mussels (data not shown).

### Biochemical results

**Figure 3:**
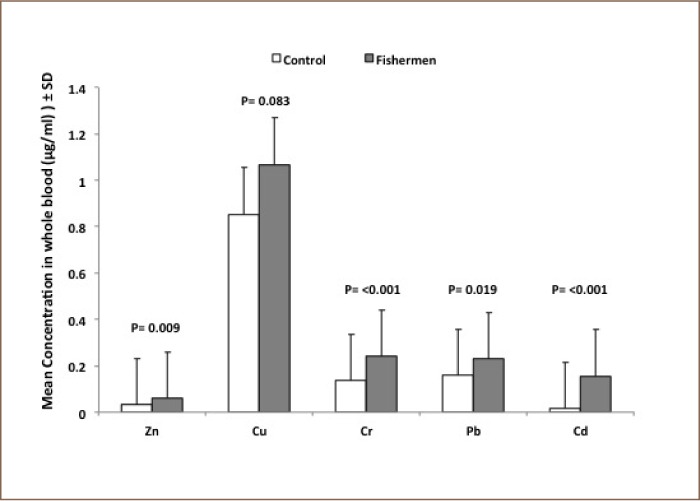
Whole blood concentrations of cadmium (Cd), lead (Pb), chromium (Cr), copper (Cu) and zinc (Zn) of the healthy control group (n = 10) and the fishermen group (n = 40)

Mean blood concentration levels of the five studied metals, Cd, Pb, Cr, Cu and Zn were 0.017±0.009, 0.159±0.04, 0.137±0.025, 0.854±0.294, 0.038±0.016 μg/ml, respectively, for the control group and 0.155±0.073, 0.231±0.081, 0.242±0.053, 1.07±0.319, 0.061±0.026 μg/ml, respectively, for the study group (*Figure 3*). Mean concentrations of the five studied metals were significantly elevated in the study group compared to control group, except for Cu which was not significantly elevated in fishermen (p = 0.083). The mean concentrations of serum malondialdehyde for the healthy control and fishermen groups are shown in Table 1. Statistical analysis using independent t-test demonstrated that there was a highly significant increase in the serum malondialdehyde levels of the study group compared to the control group (p < 0.001). On the other hand, Table 2 demonstrates the presence of a highly significant decrease in the enzymatic activities of glutathione peroxidase in the blood of the study group compared to the control group (p < 0.001). In addition, the erythrocytes superoxide dismutase activity presented in Table 3 showed no significant differences between the two groups (p = 0.462).

**Table 1:**
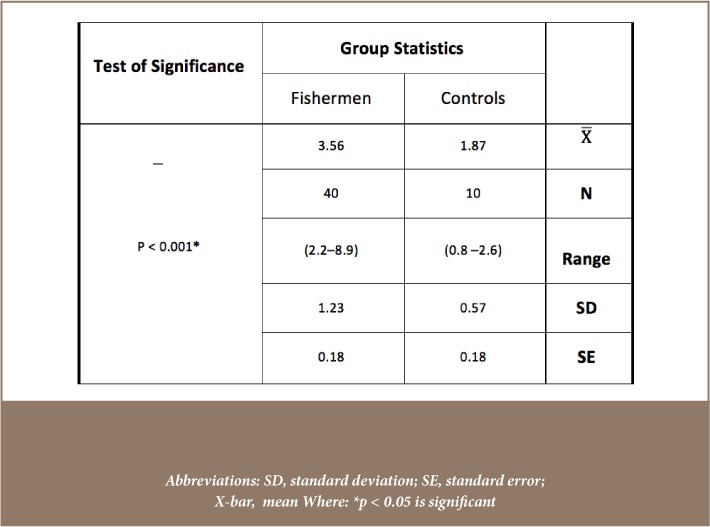
Statistical Analysis (Independent t-test) of Serum Malondialdehyde Concentrations (nM/ml) of the Healthy Control and Fishermen Group

**Table 2:**
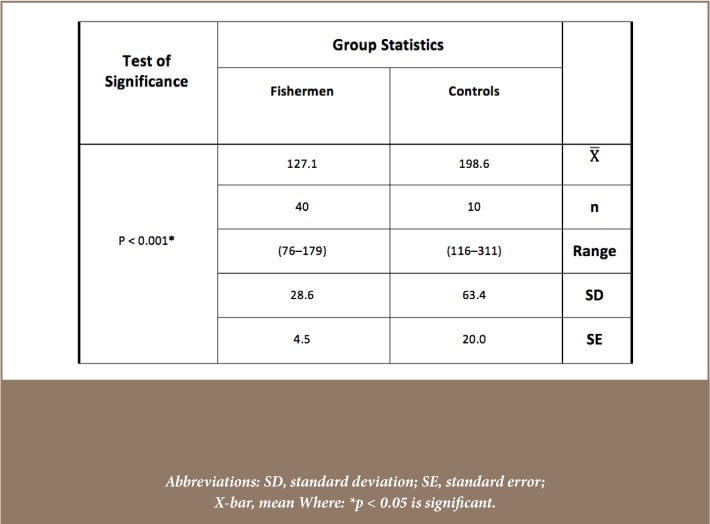
Statistical Analysis (Independent t-test) of Erythrocytes Glutathione Peroxidase Enzymatic Activity Levels (U/g Hb) Between Healthy Control and Fishermen Group

**Table 3:**
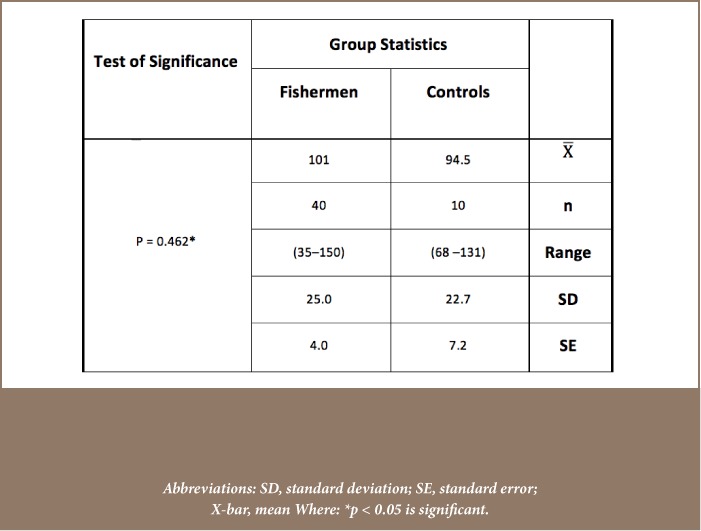
Statistical Analysis (Independent t-test) of Erythrocytes Superoxide Dismutase Enzymatic Activity Levels (U/g Hb) of Healthy Control and Fishermen Group

### Insulin-like growth factor II gene expression

**Figure 4:**
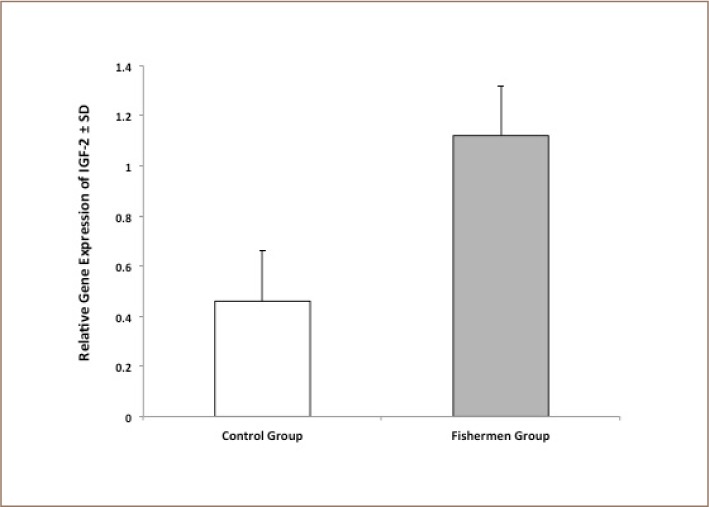
Mean relative gene expression levels of insulin-like growth factor 2 (IGF-2; expressed in ng/μl) for the healthy control and fishermen group. Error bars represent standard deviation

**Figure 5:**
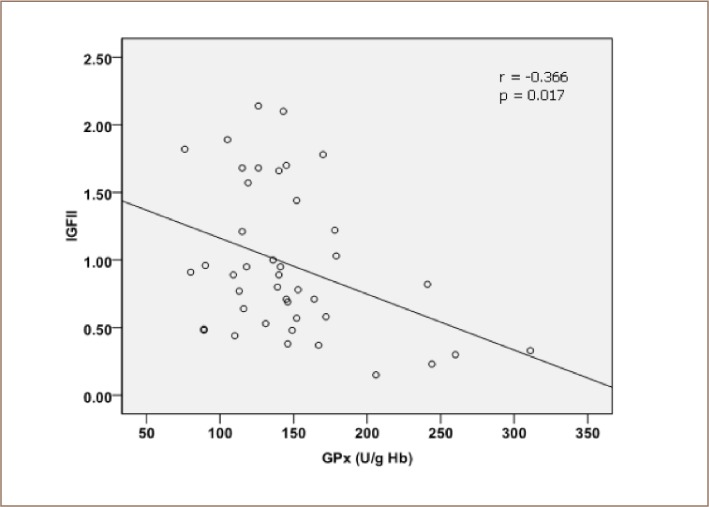
Pearson correlation between expression of insulin-like growth factor 2 gene (IGF-2; expressed in ng/μl) and erythrocytes glutathione peroxidase enzymatic activity (GPx) in fishermen

Table 4 and Figure 4 depict the relative IGF-2 gene expression levels of the control group and fishermen group. Table 4 presents the results of the statistical analysis (independent t-test) of relative gene expression levels of IGF-2. There was a highly significant increase in IGF-2 gene expression in the study group compared to the control group (p < 0.001). Figure 5 illustrates the correlation coefficient between IGF-2 gene expression and glutathione peroxidase in fishermen (r = 0.366, p = 0.017; by Pearson's linear regression analysis).

**Table 4:**
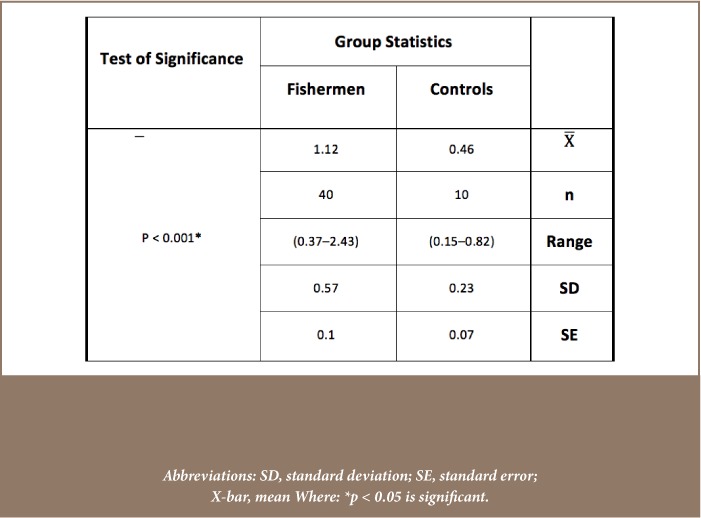
Statistical Analysis (Independent t-test) of Relative Gene Expression Levels of IGF-2 (expressed in ng/μl) Between Healthy Control and Fishermen Group

## Discussion

Contamination of the aquatic environment by heavy metals can be evaluated by analyzing water, sediments and aquatic organisms.^24^ The levels of heavy metals are often considerably higher in mollusks and other invertebrates than in other species in aquatic environments.^8–10^ Therefore; mussels are frequently used as a model for biomarker investigations because of their wide geographical distribution and the irresistance to different concentrations of heavy metals.^13^ Metallothionein is considered a specific biomarker for pollution in aquatic environments used to monitor exposure to heavy metals.^25^

The present results indicated that the study area is contaminated with heavy metals such as Cd, Pb, Cu, Cr, and Zn, confirming previous findings.^11^ The present study also confirmed the presence of measurable amounts of metallothionein in mussels collected from the study area. As heavy metals accumulate in ecosystem components (e.g. air, soil, and water), the risk of human exposure increases among industrial workers and people living near contaminated areas.^15^ Furthermore, the results of the current study demonstrate the presence of high concentrations of heavy metals such as Cd, Cr, Pb, Cu and Zn in the blood of fishermen from the El-Maadiya region. This heavy metals load in blood provokes severe oxidative stress manifested by the presence of elevated levels of malondialdehyde, and a dramatic decrease in antioxidants (i.e. decrease in antioxidant enzymes activities of glutathione peroxidase). The presence of elevated levels of heavy metals in fishermen in the present study may be due to the food habits of this group, since they consume a great deal of mussels, as reported in study questionnaires, or to another unmeasured factor. However, present smokers or those with a history of smoking were excluded from enrollment in this study.

The oxidative stress induced by heavy metals has mutagenic and carcinogenic effects on living organisms. They induce reactive oxygen and nitrogen species in mammalian cells either in vivo or in vitro.^2^ These induced free radicals are known to cause oxidative damage to lipids, proteins and DNA.^2^ In addition, they act as mitogenic signals and activate a redox state through sensitive transcription factors.^26^

The results of the present study revealed a highly significant increase in the gene expression of IGF-2 in the blood of fishermen. Insulin-like growth factor II has a structural similarity to proinsulin, IGF-1, and relaxin. Thus, IGF-2 plays an essential role in the regulation of liver cell growth and metabolism. Both IGF peptides have mitogenic, antiapoptotic and differentiation-related signaling properties. IGF-2 is highly expressed in different human and rodent fetal tissues such as the liver, kidney, and skeletal muscle, while it is down regulated or suppressed in the organs of adults. In addition, the concentration of circulating IGF-2, which arises from the liver, is correlated to hepatic integrity.^27^

Baddour et al. observed a significant positive correlation between IGF-2 expression and the inflammatory stage.^28^ This is in accordance with the findings of Grisham, which revealed that an increased IGF-2 level results from the combined actions of cytokines produced by chronic inflammatory cells from damaged liver and viral transactivation in chronic hepatitis.^29^ In this respect, the findings of the present study suggest that the investigated fishermen group may suffer from progressive hepatitis, seriously affecting their health. Recently, investigations suggested the substantial clinical significance of evaluating IGF-2 in cases of liver cancer.^30^ As a consequence, upregulation of IGF-2 gene expression has been correlated with increased rates of cell mitotic activity, as evaluated by proliferating cell nuclear antigen expression and may contribute to tumoral angiogenesis.^31^,^32^ Similarly, it has been shown that IGF-2 gene expression is stimulated during hepatocarcinogenesis in transgenic mice, and is associated with increased replication activity.^32^ Meanwhile, the present data emphasized a significant increase in the gene expression of IGF-2, with a positive correlation between expression of IGF-2 and MDA. In addition, there was a negative correlation between the gene expression of IGF-2 and the enzymatic activities of glutathione peroxidase. These combined effects lead to a manifestation of severe oxidative stress.

### Study limitations

This study includes only male control or test group subjects. This is a study limitation since the results were not also analyzed for females. It is challenging in our community to recruit female subjects due to traditions that make it difficult to obtain study consent from females.

## Conclusions

The current study determined that the fishermen group is exposed to heavy metals as evidenced by the presence of Cd, Cr, Cu, Pb and Zn in their blood. Exposure to heavy metals generates severe oxidative stress as manifested by the presence of high levels of MDA, accompanied by a marked decrease in antioxidant defense. In addition, there was a highly significant increase in the expression of the IGF-2 gene, and a positive correlation between expression of IGF-2 and MDA level, as well as a negative correlation between IGF-2 gene expression and GPx enzymatic activity.

## References

[1] Bertini I, Cavallaro G (2008). Metals in the “omics” world: copper homeostasis and cytochrome c oxidase assembly in a new light. J Biol Inorg Chem [Internet].

[2] Rolfs A, Hediger MA (1999). Metal ion transporters in mammals: structure, function and pathological implications. J Physiol [Internet].

[3] Valko M, Rhodes CJ, Moncol J, Izakovic M, Mazur M (2006). Free radicals, metals and antioxidants in oxidative stress-induced cancer. Chem Biol Interact [Internet].

[4] Evan GI, Vousden KH (2001). Proliferation, cell cycle and apoptosis in cancer. Nature [Internet].

[5] Jomova K, Volko M (2011). Advances in metal-induced oxidative stress and human disease. Toxicology [Internet].

[6] Witkiewicz-Kucharczyk A, Bal W (2006). Damage of zinc fingers in DNA repair proteins, a novel molecular mechanism in carcinogenesis. Toxicol Lett [Internet].

[7] ElNemr A, Khaled A, Moneer AA, ElSikaily A (2012). Risk probability due to heavy metals in bivalve from Egyptian Mediterranean coast. Egyptian J Aquat Res [Internet].

[8] Canli M, Atli G (2003). The relationships between heavy metal (Cd, Cr, Cu, Fe, Pb, Zn) levels and the size of six Mediterranean fish species. Environ Pollut [Internet].

[9] Sun J, Rong J, Zheng Y, Ma D, Lan X (2011). Risk assessment of heavy metal contaminated Dagu River sediments. Procedia Environ Sci [Internet].

[10] El Nemr AM, ElSikaily A, Khaled A (2007). Total and leachable heavy metals in muddy and sandy sediments of Egyptian coast along Mediterranean Sea. Environ Monit Assess [Internet].

[11] El Sikaily A, Khaled A, El Nemr A (2004). Heavy metals monitoring using bivalves from Mediterranean Sea and Red Sea. Environl Monit Assess [Internet].

[12] Hamed MA, Emara AM (2006). Marine mollusks as biomonitors for heavy metal levels in the Gulf of Suez, Red Sea. J Mar Sys [Internet].

[13] El Nemr A (2003). Assessment of heavy metal pollution in surface muddy sediments of lake Burullus, south eastern Mediterranean, Egypt. Egypt J Aquat Biol Fish [Internet].

[14] Nye MD, Hoyo C, Murphy SK (2015). In vitro lead exposure changes DNA methylation and expression of IGF2 and PEG1/MEST. Toxicol In Vitro [Internet].

[15] Dahab OA (1989). Chromium biogeochemical cycle in Abu Kir Bay, east of Alexandria, Egypt. Estuar Coastal Shelf Sci [Internet].

[16] Viarengo A, Ponzano E, Dondero F, Fabbri R (1997). A simple spectrophotometric method for metallothionein evaluation in marine organisms: an application to Mediterranean and Antarctic molluscs. Marine Environ Res [Internet].

[17] Christensen JM, Poulsen OM, Anglov T (1992). Protocol for the design and interpretation of method evaluation in atomic absorption spectrometric analysis. Application to the determination of lead and manganese in blood. J Anal Atomic Spectrom [Internet].

[18] Zeneli L, Daci NH, Daci-Ajvazi MN, Pacarizi H (2008). Effects of pollution on lead and cadmium concentration and correlation with biochemical parameters in blood of human population nearby Kosovo thermo power plants. Am J Biochem Biotechnol [Internet].

[19] Draper HH, Hadley M (1990). Malondialdehyde determination as index of lipid peroxidation. Methods Enzymol [Internet].

[20] Paglia DE, Valentine WN (1967). Studies on quantitative and qualitative characterization of erythrocyte glutathione peroxidase. J Lab Clin Med.

[21] Hopkins J, Tudhope GR (1973). Glutathione peroxidase in human red cells in health and disease. Br J Haematol [Internet].

[22] Drabkin DL (1946). Spectrophotometric studies; the crystallographic and optical properties of the hemoglobin of man in comparison with those of other species. J Biol Chem.

[23] Dong ZZ, Yao DF, Yao DB, Wu XH, Wu W, Qiu LW, Jiang DR, Zhu JH, Meng XY (2005). Expression and alteration of insulin-like growth factor II-messenger RNA in hepatoma tissues and peripheral blood of patients with hepatocellular carcinoma. World J Gastroenterol [Internet].

[24] Rzymski P, Tomczyk K, Rzymski P, Poniedzialek B, Opala T, Wilczak M (2015). Impact of heavy metals on the female reproductive system. Ann Agric Environ Med [Internet].

[25] Amiard JC, Amiard-Triquet C, Barka S, Pellerin J, Rainbow PS (2006). Metallothioneins in aquatic invertebrates: their role in metal detoxification and their use as biomarkers. Aquat Toxicol [Internet].

[26] Genestra M (2007). Oxyl radicals, redox-sensitive signalling cascades and antioxidants. Cell Signal [Internet].

[27] Sedlaczek N, Hasilik A, Neuhaus P, Schuppan D, Herbst H (2003). Focal overexpression of insulin-like growth factor 2 by hepatocytes and cholaniocytes in viral liver cirrhosis. Br J Cancer [Internet].

[28] Baddour NM, Zeid A, Farrag E, Taher Y (2011). Possible role of P21 RAS and IGF2 in the pathogenesis of hepatocellular carcinoma occurring on top of HCV. Hepatol Int.

[29] Grisham JW, Coleman WB, Tsongalis GJ (c2002). Molecular genetic alterations in primary hepatocellular neoplasms. Molecular basis of human cancer.

[30] Couvert P, Carrie A, Tezenas du Montcel S, Vaysse J, Sutton A, Barget N, Trinchet JC, Beaugrand M, Ganne N, Giral P, Chelly J (2012). Insulin-like growth factor 2 gene methylation in peripheral blood mononuclear cells of patients with hepatitis C related cirrhosis or hepatocellular carcinoma. Clin Res HepatolGastroenterol [Internet].

[31] Nardone G, Romano M, Calabro A, Pedone PV, de Sio I, Persico M, Budillon G, Bruni CB, Riccio A, Zarrilli R (1996). Activation of fetal promoters of insulinlike growth factors II gene in hepatitis C virus-related chronic hepatitis, cirrhosis, and hepatocellular carcinoma. Hepatology [Internet].

[32] Alexia C, Fallot G, Lasfer M, Schweizer-Groyer G, Groyer A (2004). An evaluation of the role of insulin-like growth factors (IGF) and of type-I IGF receptor signalling in hepatocarcinogenesis and in the resistance of hepatocarcinoma cells against drug-induced apoptosis. Biochem Pharmacol [Internet].

